# X-ray structure of full-length human RuvB-Like 2 – mechanistic insights into coupling between ATP binding and mechanical action

**DOI:** 10.1038/s41598-018-31997-z

**Published:** 2018-09-13

**Authors:** Sara T. N. Silva, José A. Brito, Rocío Arranz, Carlos Óscar S. Sorzano, Christine Ebel, James Doutch, Mark D. Tully, José-María Carazo, José L. Carrascosa, Pedro M. Matias, Tiago M. Bandeiras

**Affiliations:** 1 Instituto de Tecnologia Química e Biológica António Xavier, Universidade Nova de Lisboa, Av. da República, 2780-157 Oeiras, Portugal; 2 iBET, Instituto de Biologia Experimental e Tecnológica, Apartado 12, 2780-901 Oeiras, Portugal; 3 Department of Structure of Macromolecules, Centro Nacional de Biotecnología (CNB-CSIC), Campus Cantoblanco, 28049 Madrid, Spain; 4 Institut de Biologie Structurale (IBS), Univ. Grenoble Alpes, CNRS, CEA, 71 avenue des Martyrs CS 10090, 38044 Grenoble, France; 5 ISIS Pulsed Neutron and Muon Source, STFC, Harwell Science and Innovation Campus, Didcot, OX11 0QX UK; 6 European Synchrotron Radiation Facility (ESRF), Grenoble, France

## Abstract

RuvB-Like transcription factors function in cell cycle regulation, development and human disease, such as cancer and heart hyperplasia. The mechanisms that regulate adenosine triphosphate (ATP)-dependent activity, oligomerization and post-translational modifications in this family of enzymes are yet unknown. We present the first crystallographic structure of full-length human RuvBL2 which provides novel insights into its mechanistic action and biology. The ring-shaped hexameric RuvBL2 structure presented here resolves for the first time the mobile domain II of the human protein, which is responsible for protein-protein interactions and ATPase activity regulation. Structural analysis suggests how ATP binding may lead to domain II motion through interactions with conserved N-terminal loop histidine residues. Furthermore, a comparison between *hs*RuvBL1 and 2 shows differences in surface charge distribution that may account for previously described differences in regulation. Analytical ultracentrifugation and cryo electron microscopy analyses performed on *hs*RuvBL2 highlight an oligomer plasticity that possibly reflects different physiological conformations of the protein in the cell, as well as that single-stranded DNA (ssDNA) can promote the oligomerization of monomeric *hs*RuvBL2. Based on these findings, we propose a mechanism for ATP binding and domain II conformational change coupling.

## Introduction

RuvB-Like (RuvBL) proteins are found in the domains *Archaea* and *Eukarya*, including humans and fungi, where they exert roles in transcription regulation, DNA damage repair, cell cycle control, stress adaptation and disease^1–3^. The RuvB-Like family includes two homologous members, RuvBL1 and RuvBL2. RuvBLs assemble as heteromeric complexes that form an integral component of chromatin remodeling complexes TIP60^4^, INO80^5^ and SWR1/SRCAP^6^ and of the R2TP chaperone complex^7^. The RuvBL1/2 complex functions as chaperone itself during the biogenesis of Telomerase, H/ACA RNPs and other supramolecular complexes^2,4,8,9^. As constituents of chromatin remodeling machines, RuvBL1 and RuvBL2 are involved in epigenetic regulation of transcription through the deposition of H2A.Z in nucleosomes flanking nucleosome-depleted regions around promoters (SWR1)^6^, by remodeling nucleosomes in order to increase DNA accessibility for the transcription and repair machinery, by enabling progression of stalled replication forks (INO80) and by the acetylation of histones H2A and H4, increasing chromatin accessibility (TIP60)^4,10^. Conversely, RuvBL1 and RuvBL2 function independently and even antagonistically in many instances^11–13^. One example is during hypoxia, in which case methylation of either RuvBL1 or RuvBL2 by G9a methyltransferase leads, respectively, to the hyperactivation^13^ or downregulation^12^ of a different set of hypoxia related genes. In this case, methylation of RuvBL2 during hypoxia leads to its interaction with HIF-1α, and consequent HDAC1-dependent repression of genes such as pro-apoptotic *BNIP3* and angiogenesis promoters *PGK1* and *VEGF1*^12^. As another example, RuvBL1 can be methylated by the PRMT5 arginine methyltransferase. This was shown to be critical for the acetyltransferase activity of TIP60, and leads to H4K16 acetylation, which facilitates 53BP1 displacement from DSBs, thus regulating homologous recombination^14^. Besides regulating the cell response to stress conditions, RuvBL1 and RuvBL2 have known implications in cancer. In cancer cells, expression of the metastasis suppressor KAI1 is regulated by binding of either Tip60:RuvBL1 or β-catenin:RuvBL2^SUMO^ to its promoter. These mutually exclusive processes are directly correlated with RuvBL2 SUMOylation state, and lead respectively to upregulation or repression of KAI1 expression^15^. Moreover, both *RuvBL1*^−/−^ and *RuvBL2*^−/−^ mice are embryonic lethal^16,17^, suggesting a pivotal role for these proteins at the onset of development.

These observations led us to investigate the structural basis for their antagonistic activities. Available atomic resolution structures of RuvB-Like proteins include the human RuvBL1 hexamer^18^, the human RuvBL2 hexameric ATPase core^19^, the RuvBL1:RuvBL2 complex from the thermophilic fungus *Chaetomium thermophilum*^20,21^, and the human RuvBL1ΔDII:RuvBL2ΔDII dodecameric complex^22^. In the latter, the mobile domain II was removed for crystallization purposes. The absence of a complete structure of human RuvBL2 poses a serious limitation to the study of its function, since the truncated domain II is fundamental for activity regulation^22^. Furthermore, mutations in this domain can lead to serious organ developmental defects, such as heart hyperplasia caused by the *lik* mutant of RuvBL2. Growing interest on the multiple activities of these proteins has justified the recent inception of a multidisciplinary biennial workshop centered on the study of the multiple cellular roles in which they are involved^23,24^. In cancer patients, RuvBL2 overexpression is considered a mark of poor prognosis, and it has been suggested as a potential anti-cancer drug target^25,26^.

Knowledge on the human RuvBL2 structure and nucleotide-dependent activity is, hence, essential, as a first step towards a targeted approach to drug development. In this work, we set out to analyze the structure and function of human full-length RuvBL2 (*hs*RuvBL2). We herein present the crystallographic structure of *hs*RuvBL2 to 2.8 Å resolution, and compare it with the previously described RuvBL2 from *Chaetomium thermophilum*, and the domain II-truncated human RuvBL2 structure. Analysis of the N-termini and nucleotide binding pockets of the three proteins suggests a mechanism of chemo-mechanical coupling between nucleotide binding and domain II motion. We further tackle the interference of purification tags on the oligomerization state of the protein, which has for long been an open question in human RuvBLs, and shed some light on the mode of ssDNA binding.

## Results

### Structure of full-length human RuvB-Like 2

**Figure 1:**
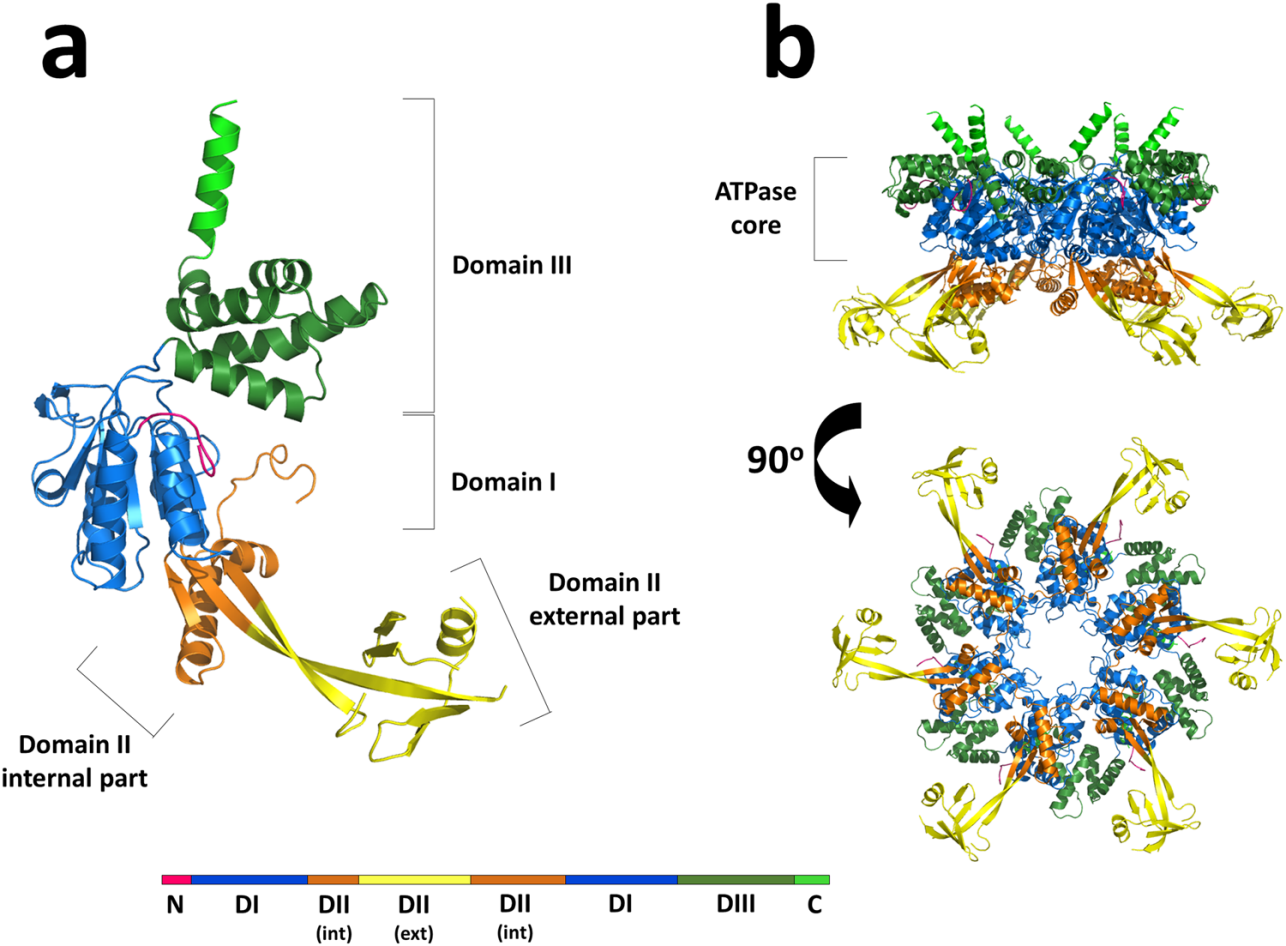
Cartoon representations of the overall structure of *hs*RuvBL2 monomer and hexamer. (**a**) The full–length apo *hs*RuvBL2 monomer. Domains I, II and III are coloured blue, orange/yellow and green, respectively. The visible part of the N-terminal loop and the C-terminal helix are also identified in pink and light green, respectively. A linear schematic representation of the domains of *hs*RuvBL2, using the same colours, is shown below, highlighting the internal and external portions of domain II. (**b**) Side and bottom views of the *hs*RuvBL2 hexamer, highlighting the AAA+ core, using the same colour code.

The crystal structure of the full-length human transcription regulation factor RuvB-Like 2 (*hs*RuvBL2) in its apo form was determined to 2.89 Å resolution (Table 1 and Fig. 1). The structure shows a hexameric arrangement of monomers similar to other structures in the RuvB-Like family^18,20–22^. The hexameric arrangement of *hs*RuvBL2 in solution was confirmed by small angle X-ray scattering (SAXS - see below) coupled to size exclusion chromatography, which unequivocally showed the formation of a complex with a mean radius of gyration of *ca*. 52 Å throughout the elution peak (Supplementary Fig. S1).

**Table 1:** Data collection and refinement statistics

Data collection		
Beamline	Proxima-1 (SOLEIL – Paris – France)	
Wavelength (Å)	0.9762	
Space group	*P* 6	
PDB entry	—	6H7X
	347°-wedge	61°-wedge
Cell dimensions		
*a*, *b*, *c* (Å)	122.97–122.97–60.84	123.02 123.02 60.88
*α*, *β*, *γ* (°)	90 90 120	90 90 120
Resolution (Å)	106.5–2.80 (2.81–2.80)	52.86–2.89 (2.94–2.89)
*R*_merge_ (%)	38.6 (125.3)	11.1 (66.0)
*R*_p.i.m._ (%)	9.2 (44.1)	6.3 (37.6)
<*I*/*σ*(*I*)>	14.4 (2.6)	11.3 (2.3)
Completeness (%)	100 (100)	97.0 (99.7)
Redundancy	16.4 (9.0)	3.9 (3.9)
*CC*_½_ (%)	96.0 (74.3)	99.6 (77.3)
Wilson *B* factor (Å^2^)	53.9	50.8
**Refinement**		
Resolution (Å)		40.27–2.89 (3.18–2.89)
No. of unique reflections		11274
R_work_/R_free_ (%)		20.5/23.7 (26.8/31.2)
No. Atoms		
Protein		2884
Water		40
*B* factors (A^2^)		
Average		58.4
Protein		58.6
Solvent		38.31
r.m.s. deviations		
Bond length (Å)		0.002
Bond angles (°)		0.424
Ramachandran plot		
Favoured		97.5
Outliers		0.0
Rotamer outliers (%)		0.95
Clashscore		3.42
MolProbity score		1.23

Values in parenthesis refer to the highest resolution shell.

In the ring-shaped *hs*RuvBL2 hexamer, each protomer comprises the typical Rossman-like αβα fold (domain I) and the canonical all-α subdomain (domain III), which form the ATPase core together with the internal region of domain II. Moreover, there is an outward-facing mobile unit composing the external region of domain II (Fig. 1), linked to the ATPase core by two β-sheet linkers. This unit is absent in all previous crystal structures of human RuvBL2 and is observed here for the first time. The interfaces between protomers form a complete nucleotide binding pocket, which includes the canonical nucleotide-binding motifs in domains I and III: Walker A (G77-T84), Walker B (D299-H302), sensor 1 (M326-N329), sensor 2 (T397-A402) and a trans-arginine finger (R353) from the adjacent protomer. Despite the fact that all purification steps were performed in the presence of ADP, no electron density corresponding to this nucleotide (or segments thereof), could be observed in the pocket. Protruding from the C-termini of each protomer in the hexameric-arranged ATPase cores there are six solvent-reaching antennae-like α-helices. Domain II, located between the Walker A and Walker B motifs of domain I, bears no significant sequence similarity to other known domains, except for residues 136–233 which, similarly to RuvBL1 residues 131–227, organise into an oligonucleotide/oligosaccharide-binding (OB)-fold^27^. Hence, domain II is unique to RuvB-Like proteins. In addition to the overall mobility of domain II, there are four additional loops that are probably too mobile to produce an electron density: D148 - G159, D185 - V187, S203 - C227 and R253 - Q255. The second and fourth segments are long and located on the external part of the domain II.

The *hs*RuvBL2 hexamer is 149 Å wide, measured using the Cα of residue Q188 as reference point (versus 147 Å in *hs*RuvBL1 measured at E212 and 120 Å in the RuvBL1:RuvBL2 dodecamer from *Chaetomium thermophilum*, which crystallized with a more compact conformation of domain II towards the ATPase core, measured between D343 of RuvBL1 and D338 of RuvBL2). The ATPase core is 51 Å high, similar to 50 Å in RuvBL1^18^ and 51 Å in the truncated dodecameric complex^22^. The *hs*RuvBL2 hexamer has a central channel 23 Å wide (similar to the *Ct*RuvBL1:RuvBL2 dimensions^20,21^, and slightly larger than the channel of *hs*RuvBL1, which is 20 Å wide) on its narrowest part and although a double-stranded B-DNA molecule could be tightly fitted (not shown), it has been clearly demonstrated biochemically that RuvBL2 can only bind single-stranded DNA^28^. To the best of our knowledge, similar studies have not been published concerning RuvBL1.

**Figure 2:**
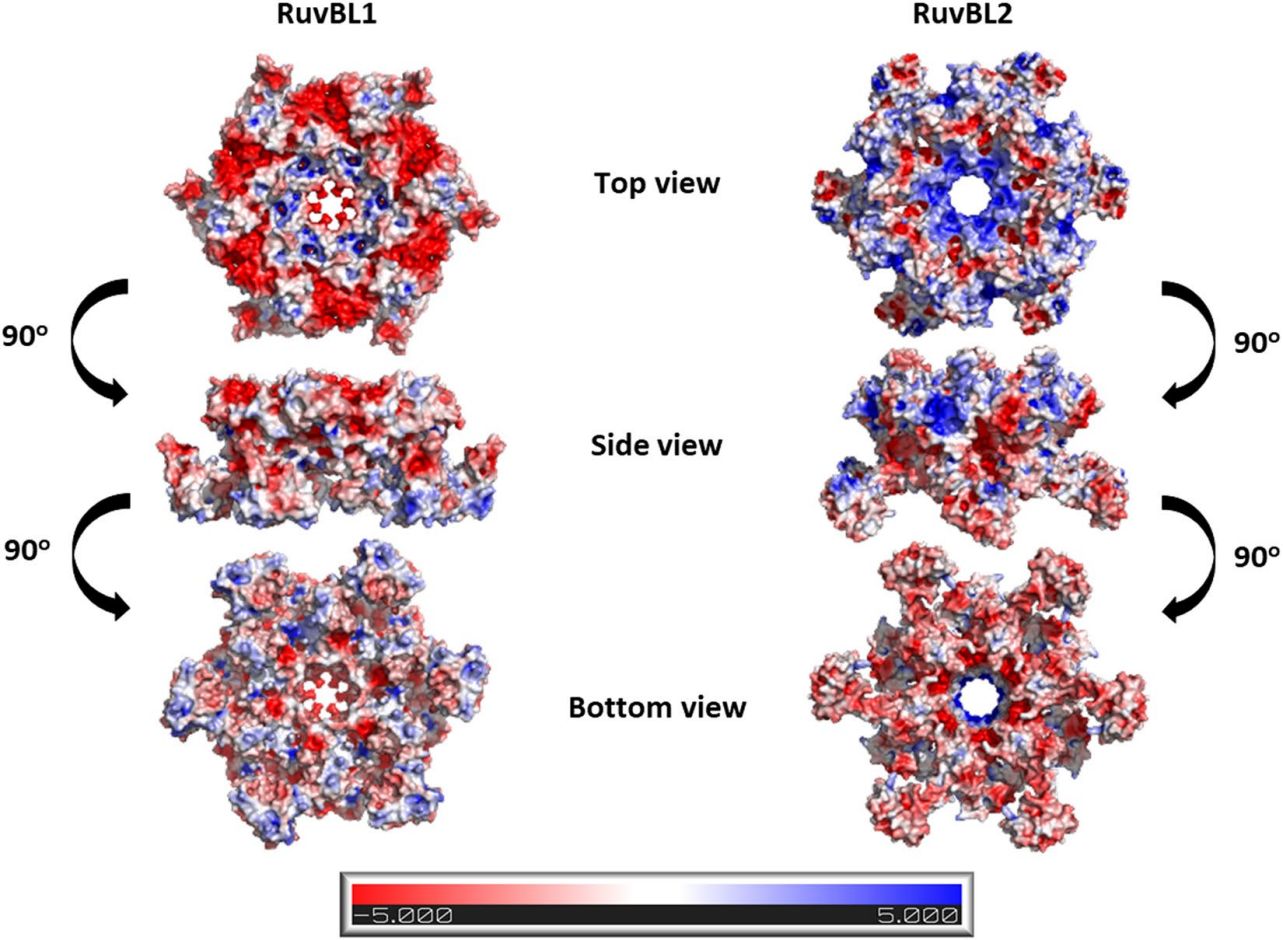
Surface charge distribution in the *hs*RuvBL1 and *hs*RuvBL2 hexamers. Differences in the distribution of amino acid residues at the surface of the rings may underlie their antagonistic activities. Colour by electrostatic potential on solvent accessible surface. Scale bar at the bottom, in kT/e.

RuvBL1 and RuvBL2 are homologous proteins with 43% sequence identity and 65% sequence similarity that work together as part of chromatin remodeling complexes^4,8,29–31^. However, they also work antagonistically in many situations. It has been suggested that the antagonistic activities of RuvBL1 and RuvBL2 may be the consequence of interactions with different, more or less specific partners^32^. Having access to the full-length structure of *hs*RuvBL2, and to assess the structural basis for these interaction specificities, we analyzed the structures of both *hs*RuvBL1 and *hs*RuvBL2 for differing characteristics at the surface of both complexes. A comparison of the surface charge distribution of *hs*RuvBL1 and *hs*RuvBL2 hexamers (Fig. 2) shows *hs*RuvBL2 to have a markedly more positive inner channel and ATPase core (with small negatively charged patches at the top), and a more negative distribution on the bottom surface of domain II. These charges are mostly inverted in *hs*RuvBL1, which suggests different mechanisms/affinities for binding DNA or other proteins. Furthermore, *hs*RuvBL1 and *hs*RuvBL2 have different oligomerization dynamics, since monomer to hexamer transition occurs at a much higher concentration for *hs*RuvBL1 than for *hs*RuvBL2 (ref.^22^ and this work). The sum of these observations suggests considerable differences in specificity.

### Structural basis for coupling ATP binding to mechanical action in RuvBL2

**Figure 3:**
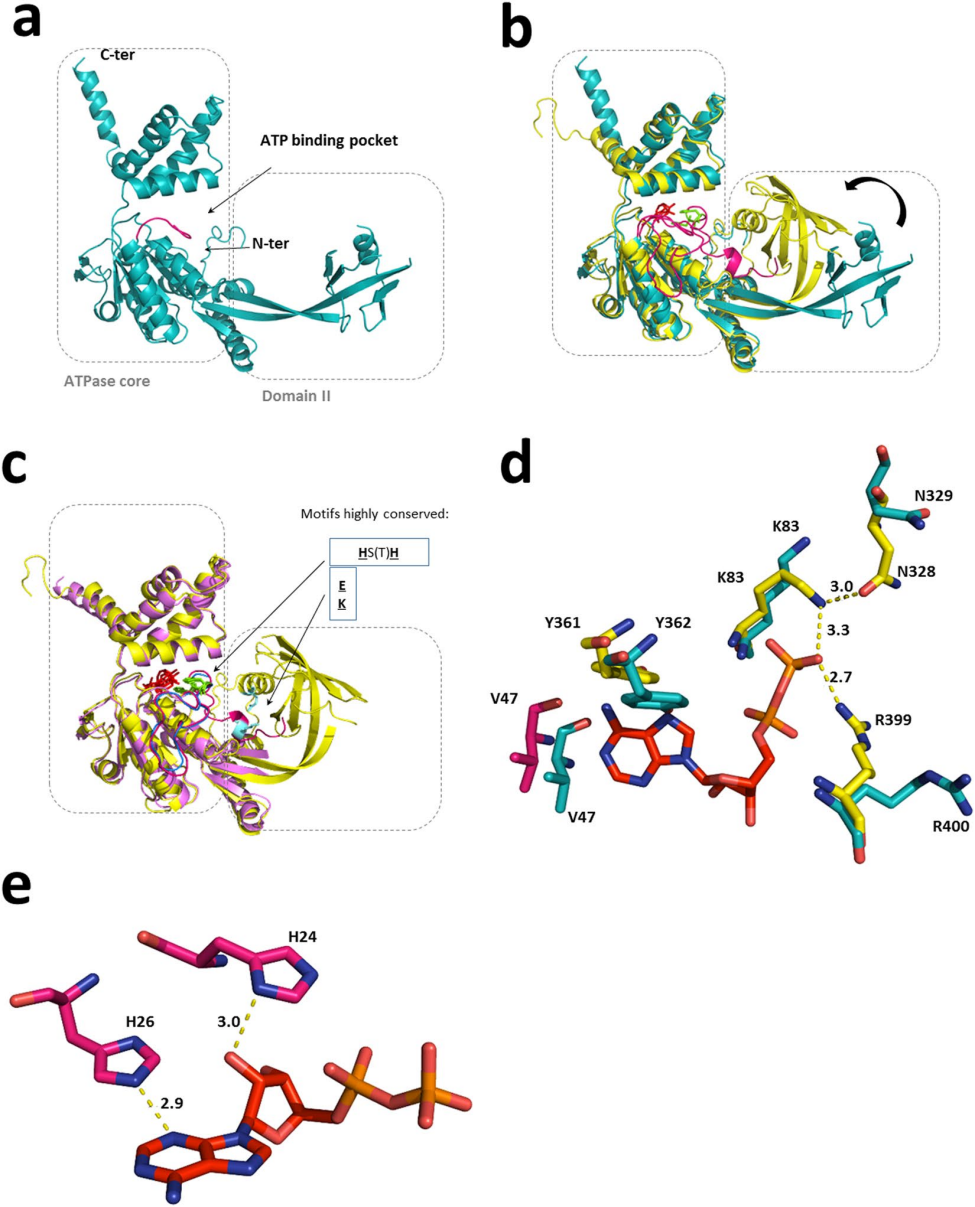
Comparison between existing structures of RuvBL2 suggests a connection between nucleotide binding and domain II motion. (**a**) The apo *hs*RuvBL2 (blue, this work, PDB ID 6H7X) is associated with a stretched, or at least loose conformation of the domain II. (**b)** The apo RuvBL2 is superimposed with the ADP-bound RuvBL2 from *Chaetomium thermophilum* (yellow, PDB ID 4WW4). When compared to the apo *hs*RuvBL2, the ADP-bound *ct*RuvBL2 displays a positioning of domain II closer to the ATPase core and a more ordered N-terminal loop (pink). ADP is depicted in red sticks. **(c)** The ATP-bound, domain II-truncated *hs*RuvBL2 (light pink, PDB ID 2XSZ) is superimposed with the full-length *ct*RuvBL2 (4WW4). It becomes apparent that the absence of the external part of domain II has an influence in the organization of the N-terminal loop. In both nucleotide-bound forms, the N-terminal loop interacts with the nucleotide through two conserved histidines; however, in the domain II-truncated *hs*RuvBL2, the N-terminal loop (blue) remains disordered from the first residue up to the place of interaction with the nucleotide. The histidines that interact with the nucleotide are depicted in green and the motif is highlighted; residues involved in electrostatic interactions between the N-terminal loop and domain II are depicted in light blue and highlighted as well. (**d**) Superimposition of the nucleotide binding pocket of *ct*RuvBL2 (yellow, 4WW4) with the human RuvBL2 homologous residues (blue, 6H7X – this structure). The ADP molecule (depicted in red) is from the fungal structure. Atoms are coloured as follows: N – dark blue; O – salmon; P – dark yellow. V47 from *C*. *thermophilum* is depicted in pink since it is part of the N-terminal loop. (**e**) Position of conserved histidines 24 and 26 in relation to ADP in the binding pocket of *Ct*RuvBL2. The interatomic distances shown are in Å.

It is a sensible assumption that the basis of ATP-dependent activities in RuvB-Like proteins lies upon the mechanical consequences of ATP binding and hydrolysis. In order to elucidate what these consequences might be in the case of RuvBL2, we compared the structure of the full-length human apo RuvBL2 (this work, PDB ID 6H7X) with the ADP-bound variant of RuvBL2 from the fungus *C*. *thermophilum*^21^ (*ct*RuvBL2, PDB ID 4WW4). Both proteins share 68% identity and 85% sequence similarity, as well as a conserved disposition of the elements that constitute the ATP binding pocket (Fig. 3d). In *ct*RuvBL2, nucleotide binding leads to a tightening of the binding pocket near the phosphate tail. The β-phosphate of the ADP molecule interacts with neighbouring residues K83, N328 and R399 of *ct*RuvBL2 (which correspond to K83, N329 and R400 of *hs*RuvBL2). Concomitantly, nucleotide entrance into the binding pocket causes a displacement of V47 and Y361 (V47 and Y362 in the human protein) due to hydrophobic interactions with the adenine ring, possibly driving a spatial rearrangement of residues in the vicinity of the nucleotide binding pocket that may be the origin of the downstream rearrangement of the N-terminal loop. We propose that these interactions initiate the coupling between nucleotide binding and mechanical movement of domain II.

While the apo *hs*RuvBL2 structure has no visible electron density up to residue 47 (N-terminus segment shown in dark pink in Fig. 3a), ADP-bound *ct*RuvBL2 (yellow in Fig. 3b) has electron density for most of the N-terminal loop, supporting a connection between ADP binding and N-terminus mobility, as previously suggested^20^. Furthermore, in ADP-bound *ct*RuvBL2, domain II is more tightly packed against the ATPase core, a conformation in which this domain interacts closely with the proximal part of the N-terminus. The apo *hs*RuvBL2 structure was further compared with the ATP-bound form in the *hs*RuvBL1ΔDII:RuvBL2ΔDII complex^22^ (Fig. 3c, light pink; noteworthy, this protein was crystallized with the domain II truncated, PDB ID 2XSZ). This alignment shows that in the absence of an interaction between the N-terminus proximal segment and the external region of domain II, the N-terminal loop is stabilized only from the point where it interacts with the bound nucleotide. This nucleotide interaction occurs through the histidine residues of a conserved **H**S**H** motif (H25 and H27 in the wild type *hs*RuvBL2, H40 and H42 in the domain II-truncated *hs*RuvBL2 and H24 and H26 in *ct*RuvBL2), while the remaining proximal part remains unstructured. The same is observed in the structure of the human DII-truncated complex associated with the RBD domain of RPAP3 (PDB ID 6FO1)^33^. These structure comparisons strongly support a connection between nucleotide binding, N-terminus rearrangement and domain II conformational stabilization in closer proximity to the ATPase core.

These observations support the proposal of the following sequential mechanistic events: in ATP-dependent activities, the nucleotide first binds to the nucleotide-binding pocket, eliciting a rearrangement of the surrounding residues, which causes the most distal part of the N-terminus to move. Video 1 (Supplementary Information) highlights the predicted movements that would occur in a RuvBL2 monomer, during its transition from the apo to the nucleotide-bound form. Note the movement of the N-terminal loop as it is tethered by interactions with the nucleotide molecule through H24 and H26 in *ct*RuvBL2. This initial interaction then draws the remaining part of the N-terminus into the close vicinity of the external region of domain II. Here, the conserved N-terminus residues E11, K13 and E14, and domain II residues K183 and I201 (E12, R14, D15 and K184 in *hs*RuvBL2) seem to be responsible for the electrostatic interactions that sustain the positioning of domain II in close proximity to the ATPase core, as seen in ADP-bound *ct*RuvBL2 (yellow in Fig. 3). Thus, our apo *hs*RuvBL2 structure provides a missing link that supports the mechanism proposal whereby the N-terminal loop provides an interface between the external region of domain II and the ATPase core. Key residues involved in the proposed mechanism are not only conserved between the human and fungal forms (Supplementary Fig. S2), but throughout the RuvBL2 family.

On the other hand, nucleotide binding to RuvBL1 does not elicit the same structural changes as for RuvBL2. In fact, despite the initial interaction of the two conserved histidines (H18 and H20 in the human RuvBL1, PDB ID 2C9O) with the nucleotide, both in human and in *C*. *thermophilum* RuvBL1 the N-terminal loop is directed towards interactions with domain I of the same protomer: K11 interacts with P95 from *hs*RuvBL1 and R15 interacts with P96 from *ct*RuvBL1.

### *hs*RuvBL2 oligomer state and stability can be modulated

**Figure 4:**
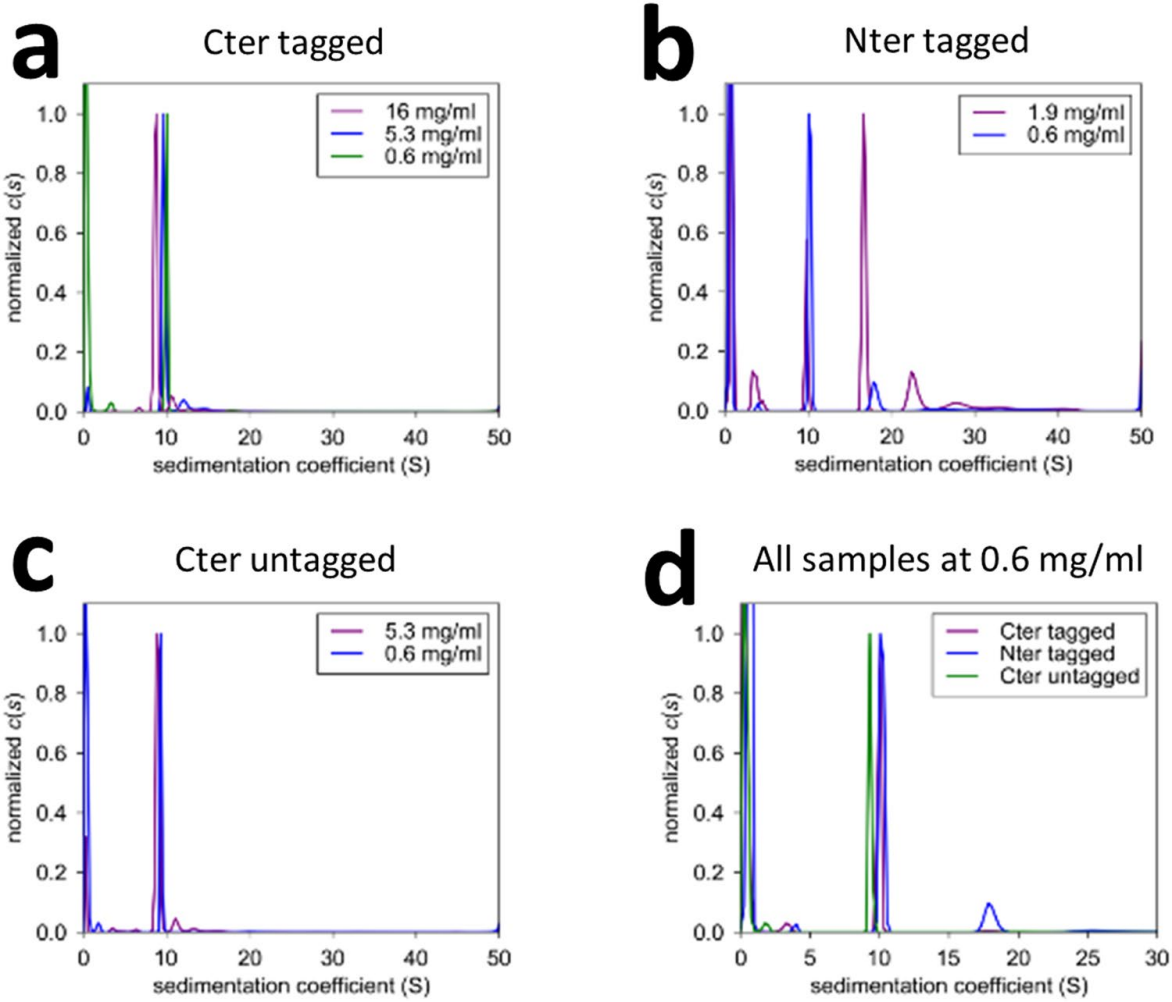
Analytical ultracentrifugation analysis of *hs*RuvBL2 samples. The oligomeric plasticity of *hs*RuvBL2 varies with the position of the tag: when placed on the C-terminus, *hs*RuvBL2 forms hexamers in all concentrations tested, even after tag cleavage (**a**) and (**c**). However, when expressed with the tag on the N-terminus, *hs*RuvBL2 oligomeric state varies with concentration (**b**), forming monomers, hexamers, dodecamers and high molecular weight species.

To address whether the location of the affinity tags would affect the oligomeric assembly and stability of human RuvBL2, we expressed recombinant *hs*RuvBL2 in *E*. *coli*, with affinity tags either at the N- or C-terminus. Purifications were performed in presence of ADP, which initial thermal shift assays (TSA) showed to provide the highest degree of thermal stability (as compared to ATP, AMP-PNP and no nucleotide). We analyzed the obtained oligomeric forms by analytical ultracentrifugation (AUC), and analyzed their stability by TSA. Tags placed at the N-terminus destabilized *hs*RuvBL2 the most, as confirmed by TSA (SM Fig. S3a), both in the native and SeMet forms, and also led to the formation of multiple oligomeric forms (SM Fig. S3b and c). Furthermore, analytical ultracentrifugation showed a concentration-dependent oligomer formation of the N-terminally tagged *hs*RuvBL2, where higher oligomeric forms and monomers appeared as concentration increased (Fig. 4b). On the other hand, C-terminally tagged *hs*RuvBL2 consistently formed hexamers at all tested concentrations (Fig. 4a). The effect of the N-terminal tag in dodecamer formation is not clear, but it may be relayed through an influence in domain II, since it has been shown that this domain is important in dodecamer stabilization in the RuvBL1:RuvBL2 complex^22,34^.

**Figure 5:**
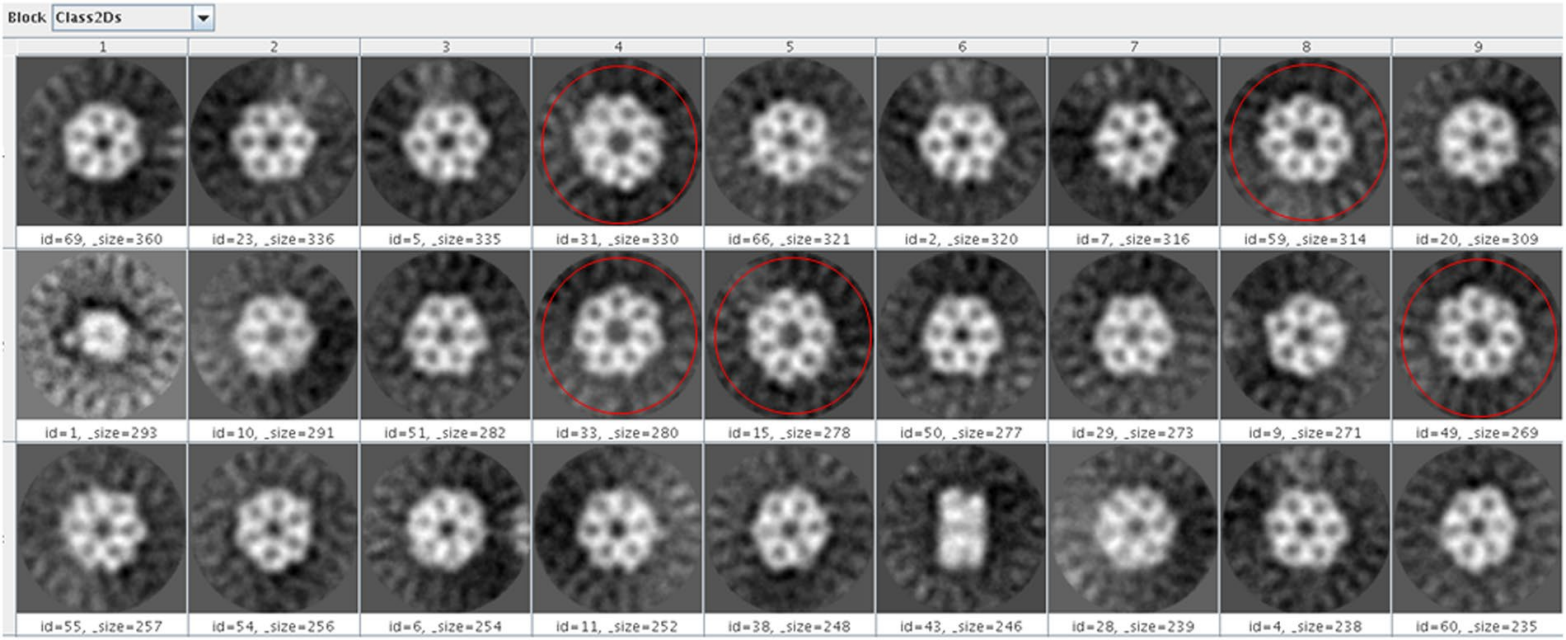
Negative staining EM analysis shows that *hs*RuvBL2 is able to form heptamers. 10% of the total of picked particles corresponded to this oligomeric form, highlighted in red.

To complement the oligomer analysis, a preliminary negative staining EM analysis was performed on the untagged *hs*RuvBL2 form. Interestingly, about 10% of particles picked from micrographs were shown to be heptamers (Fig. 5), while the remaining were hexameric rings, as expected from observations in solution. This low percentage of heptamers would have gone unnoticed by the other techniques used, since the size difference to hexamers is within their respective error margins. As far as we know, this is the first time heptamers have been observed in the RuvB-Like family. However, this oligomeric form has been observed for some AAA^+^ ATPases, such as the NtrC1 of *Aquifex aeolicus*^35^.

### *hs*RuvBL2 binding to DNA

**Figure 6:**
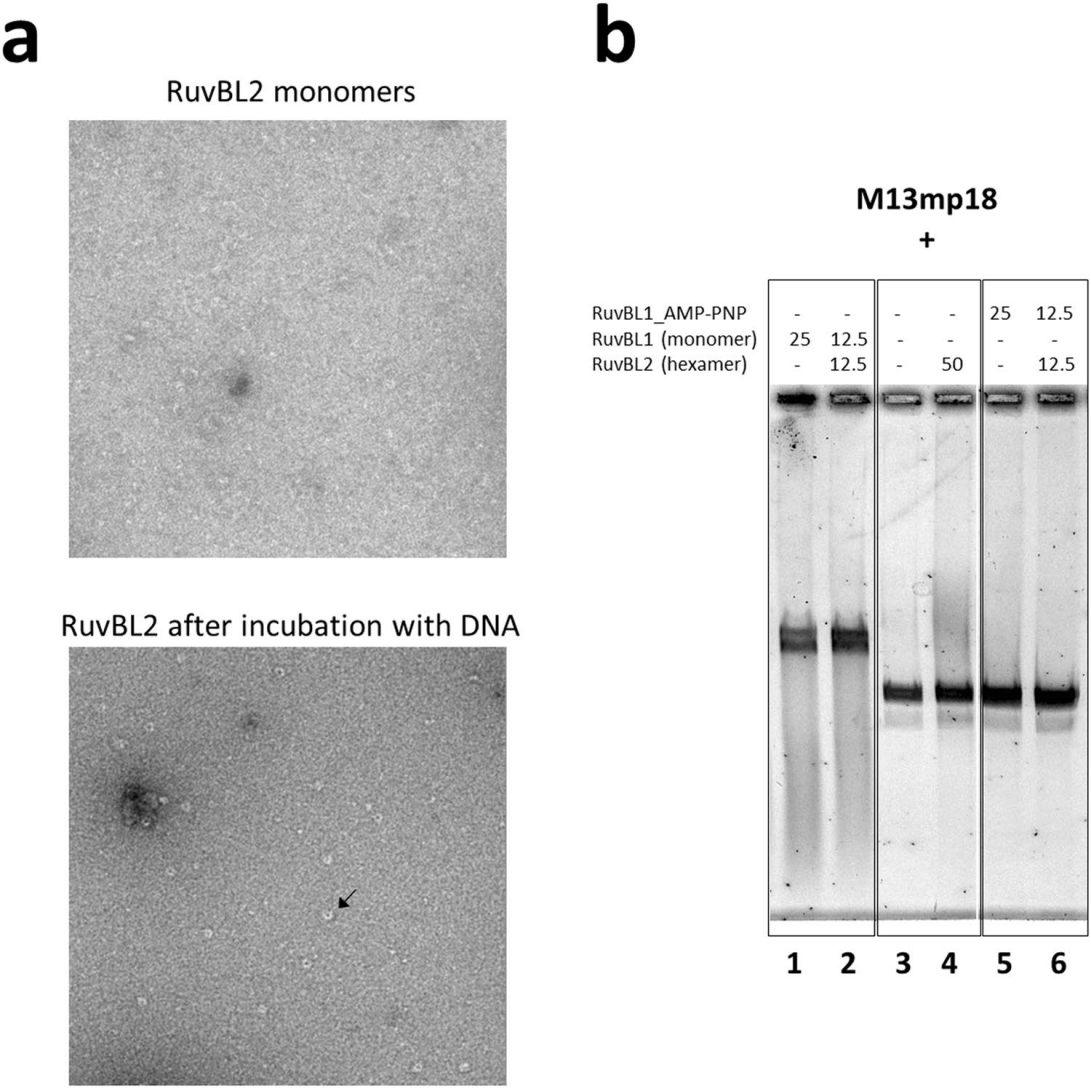
*hs*RuvBL2 interaction with DNA. (**a**) Interaction of monomeric *hs*RuvBL2 with ssDNA promotes ring reassembly, as observed by negative-staining EM. The black arrow highlights an *hs*RuvBL2 ring. An electrophoretic mobility shift assay (**b**) shows that hexameric *hs*RuvBL2 did not bind to ssDNA (lane 4), as there is no shift in running distance when comparing to the one observed by DNA alone (lane 3). However, when co-incubated in equimolar amounts with *hs*RuvBL1 (lane 2), there is a shift that indicates protein binding. Since this shift is equivalent to the one observed when incubating a total molar amount of only *hs*RuvBL1 (lane 1), we surmise that *hs*RuvBL2 is also bound to DNA in the former, most likely mediated by *hs*RuvBL1. Lanes 5 and 6 show no such binding in the presence of AMP-PNP, indicating that this non-hydrolysable nucleotide somehow prevents DNA binding.

*hs*RuvBL2 binding to DNA is independent of nucleotide sequence, and restricted to single-stranded DNA^22,28^. Papin and colleagues have also clearly shown that only monomeric RuvBL2 can bind the polynucleotide chain, which suggests a mechanism of action whereby RuvBL2 oligomerizes around a single chain overhang, and only then starts to perform the upstream unwinding of the remaining double helix, in an ATP-dependent process^28^. In order to assess what structural changes may occur upon DNA binding, we analyzed this process by negative staining EM. We first observed the dissociation of *hs*RuvBL2 hexamers into monomers after dilution to a sufficiently low concentration (*ca*. 100 µg/mL). We subsequently incubated the monomeric *hs*RuvBL2 with single-stranded DNA, and observed that the presence of ssDNA promoted the re-formation of the previously disassembled rings (Fig. 6a). The low protein concentrations used limits the number of techniques capable of detecting quaternary structure changes taking place upon interaction of *hs*RuvBL2 with DNA molecules; however, although the circular M13mp18 DNA strand used in these studies could not be observed at the resolution obtained by negative-staining EM, it can be assumed that, if the rings assembled around the DNA chain, the latter would remain associated with the protein complex. Alternatively, DNA binding may occur through the external region of domain II, as suggested for RuvBL1^18^, since both proteins display an OB-fold within this region. However, if binding occurred only through domain II, it would probably have also been observed for the hexameric forms, since this part of the protein is solvent-accessible. However, this binding mode was never observed, neither in this work, nor in previous biochemical studies performed with the human and yeast RuvBL2^28^. Furthermore, helicase activity – for which prior binding to the DNA strand is required - was shown in complexes where the external region of domain II was deleted^22^. To further address this question, we performed electrophoretic mobility shift assays (EMSA) using the same circular M13mp18 DNA (Fig. 6b). We confirmed the lack of binding of hexameric *hs*RuvBL2 to M13mp18 (lanes 3 and 4), since the observed migration length for ssDNA alone and for ssDNA in presence of hexameric *hs*RuvBL2 was the same in both lanes. Interestingly, the hexamer was able to indirectly bind DNA, in an interaction apparently mediated by monomeric *hs*RuvBL1 (lane 2) and ATP-dependent (lanes 5 and 6).

### RuvBL2 displays flexibility of domain II

**Figure 7:**
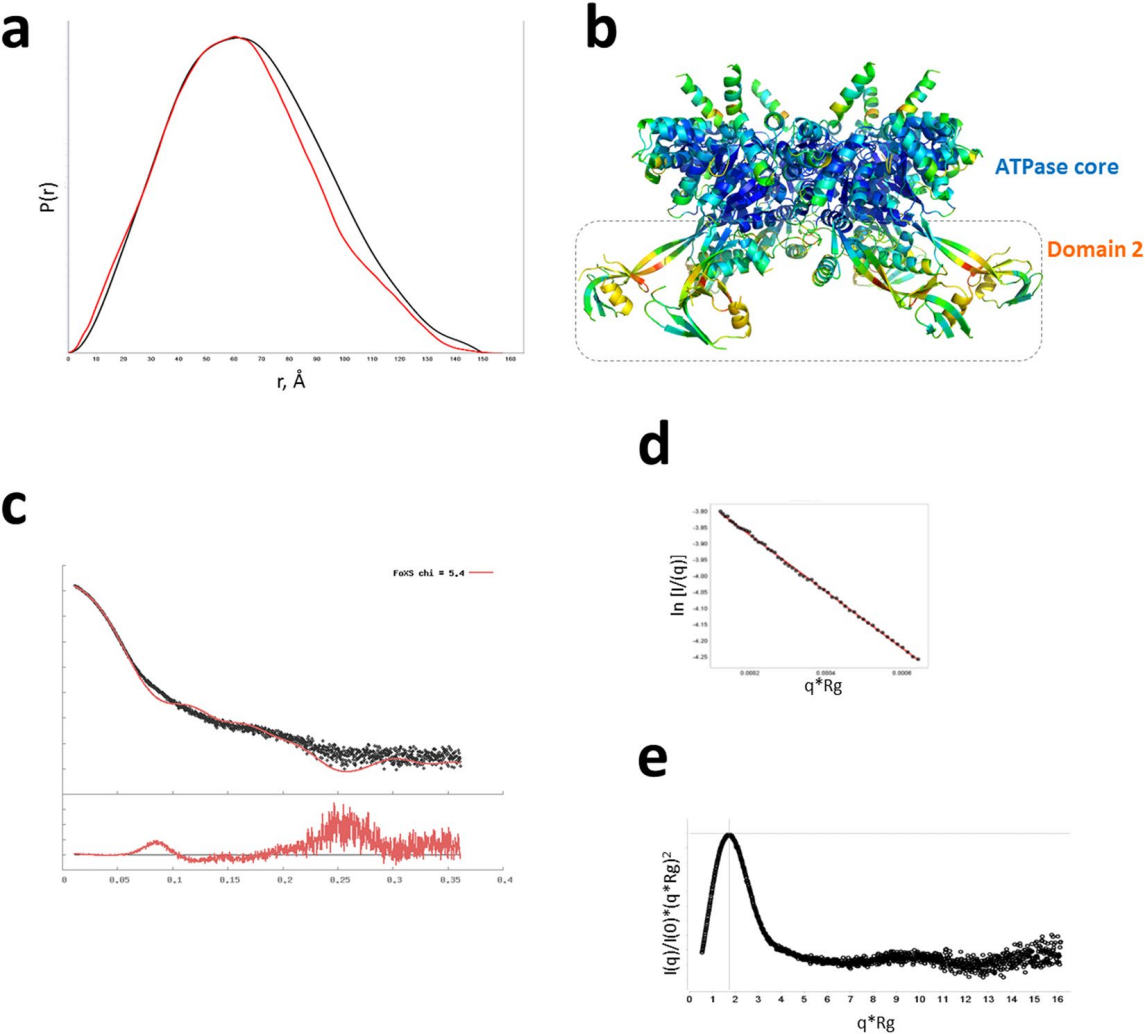
Domain flexibility of *hs*RuvBL2. (**a**) Pair distribution function, P(r) curve for experimental data (black) giving a *d*_*max*_ 150 Å overlaid with the P(r) calculated from the crystallographic coordinates (red) giving a *d*_*max*_ 157 Å. (**b**) Side view of the *hs*RuvBL2 hexamer. Atoms are coloured according to B-factor: blue – lowest; red - highest. (**c**) Small angle scattering Intensity versus scattering vector plot obtained experimentally (black), superimposed with a theoretical plot calculated from the crystallographic coordinates (red). (**d**) Guinier fit showing straight line over *q*-range. (**e**) A dimensionless Kratky plot showing the protein is globular and well folded.

Molecular dynamics studies previously showed a propensity for the external region of domain II of RuvBL hexamers to acquire a variety of conformations in solution^19^ which may be the reason behind the recalcitrant behaviour towards crystallization. In order to gain further insight into the overall shape and flexibility of RuvBL2 hexamers, we compared the pair distribution function, P(r) profile of *hs*RuvBL2 in solution (from an experimental SAXS data collection - Fig. 7a, black) with a theoretical plot calculated from the crystallographic coordinates (shown in red). The superimposed plots show that, in solution, *hs*RuvBL2 dmax is 150 Å, which is similar to that observed in the crystallographic structure of 157 Å. Comparison between the two profiles also shows an overall similar shape with the wider profile for the solution structure in the P(r) suggesting that both are globular with the protruding arms of domain II in different configurations in solution. Since d_max_ is similar, these results support a change in conformation of the protruding domains, in the hexamer, as proposed by Petukhov and colleagues^19^, lending an appearance of homogeneity of structure to the plot obtained experimentally. This mobility is further supported by the fact that the atomic B-factors are highest for the proposed β-sheet linker “hinge” residues, which form the bending point between domain II and the ATPase core (Fig. 7b).

## Discussion

We have determined the full-length crystallographic structure of human transcription factor RuvB-Like 2. The collected results suggest a putative mechanism whereby binding of a nucleotide to the binding pocket promotes the stabilization of the external part of domain II against the ATPase core, by eliciting conformational changes of the N-terminal loop. It is in fact possible that the functions of the external region of domain II may be in great part related to its interaction (mediated by the N-terminal loop) with the nucleotide in the binding pocket, with a concomitant motion with as yet undefined consequences. One of these could be the observed disassembly of dodecameric RuvBL1/2 into hexamers upon incubation with ATP^29^. It is also worth noting that the OB-fold comprises distinct superfamilies, and while the most common relates to nucleic acid-binding, they can also occur in larger proteins as recognition domains, and have even been found at protein-protein interfaces^27^. While an interaction of the OB-fold in domain II with DNA was observed for RuvBL1^18^, it is possible that the OB-fold in RuvBL2 may also be important for its interaction with other proteins in a supramolecular assembly, such as within the Ino80 complex, where cross-linking/mass spectrometry analysis shows a strong interaction profile between the OB-folds of RuvBLs and Ies2^5^. Interestingly, EM micrographs showed *hs*RuvBL2 hexamers to be frequently found in direct lateral contact, forming continuous strings (data not shown), which is also suggestive of a tendency of domain II to act as a mediator in protein-protein interactions.

Recombinant *hs*RuvBL2 has been used as a tool for *in vivo* assays, enabling the identification of binding partners such as c-myc^36^. However, affinity tags placed on the N-terminus of RuvBL1 and RuvBL2 were shown to induce the formation of dodecamers, for example, in the yeast Rvb1:Rvb2 complex^37^. The influence of tags was thus suggested to reflect the multiple conformational changes that these proteins can undergo while performing their functions, changes that *in vivo* are induced by binding partners^37^. Here we also show that the use of affinity tags in *hs*RuvBL2 expression interferes with oligomeric flexibility. Our results also seem to suggest that affinity tags may interfere negatively with *hs*RuvBL2 activity by decreasing its stability.

An analysis of the surface electrostatic charge distribution in *hs*RuvBL1 and *hs*RuvBL2 (Fig. 2) allows an overall view of distinguishing features between the two homologs that may illustrate the basis for some antagonistic roles. The smaller size of the inner channel of *hs*RuvBL1 as compared to *hs*RuvBL2 and the marked differences in the surface electrostatic charge distribution suggest the possibility of a different DNA-binding mechanism between *hs*RuvBL1 and *hs*RuvBL2. Since their activities were observed to increase when working together as a heteromeric complex^22^, it is also possible that their individual characteristics complement each other, with a consequent increase in DNA processing efficiency. Interestingly, the inner channel of the RuvBL1/2 complex, being composed of alternate positive and negative charges, may also have the function of binding peptide tails; such an interaction occurs between the ankyrin repeat domain of synphilin, which is the domain responsible for its aggregation, and K372 of RuvBL1, which is located on the surface of the ATPase core and in close proximity to the central channel^38^. The differences observed in charge distribution may also reflect distinguishing motifs that justify antagonistic activities by different interactions with protein binding partners, even when in the form of a heteromeric complex. Such an example is the preferential binding of RPAP3 C-terminal fragment to the ATPase core of *hs*RuvBL2 of a RuvBL1/2 complex^33^.

The biological significance of the *hs*RuvBL2 heptamers, if any, is still to be determined. However, it is tempting to speculate that the capacity to form heptamers could bring extra flexibility in the association to other proteins, and possibly permit an association with dsDNA, since the central channel seems to be wider.

RuvBLs have been identified as taking part in many supramolecular assemblies, where their ATPase activity is not always required. Such versatility has long posed a mystery, since no specific function can be attributed to these proteins, and yet they appear to be critical for the regulation of an array of complexes, particularly related to the control of gene expression and DNA damage response. It is possible that one of the functions of RuvBLs within larger complexes could be to recognize transient client proteins, allowing the recognition of the needs of the cell and translating them to the rest of the complex, thus regulating its activity. The fact that only heteromeric RuvBL1/2 complexes in the cell have been experimentally demonstrated to date adds another layer of complexity to their roles in transcription regulation.

In cancer patients, RuvBL2 overexpression is considered a marker of poor prognosis^25^. Previous works have shown that, in hypoxic conditions, RuvBL2 is methylated at K67 (located in domain I, in blue in Fig. 1a), which leads to downregulation of pro-apoptotic BNIP3 and pro-angiogenic phosphoglycerate kinase 1^12^. Combining these observations, it is conceivable that, when a tumor reaches hypoxic state, overexpression of RuvBL2, and its consequent methylation *en masse*, may contribute to a large-scale downregulation of the target genes. The consequence would be the already described increased resistance of hypoxic tumors to chemo and radiotherapy treatments^39^. This detailed structure of *hs*RuvBL2 can thus contribute to the development of compounds to either target the surface of RuvBL2-K67Me or aimed at the disruption of the HIF-1α:RuvBL2-K67Me complex. Further studies on distinct post-translational modifications to surface residues of RuvBLs may provide a framework for the development of compounds that can regulate the specific activities of RuvBL1 and RuvBL2, depending on the tissue, cell stage or stress conditions of the target cells. By targeting specific modifications of RuvBLs that result from particular metabolic cellular states, it might be possible to acquire the degree of specificity desired when aiming for the targeting of a specific cell subset.

## Materials and Methods

### Protein expression and purification

The codon-optimized sequence of *hs*RuvBL2 with a C-terminus-His_6_ tag including a 3C protease cleavage site was obtained from Genscript (USA), as the vector *pET49b_ruvbl2_Cter_His*, which was transformed into *E*. *coli* B834. Gene expression was induced at an OD_600_ of 0.8 by the addition of 100 µM IPTG, at 30 °C for 19 h. The cells were collected by centrifugation at 11000 × *g* for 30 minutes and disrupted in lysis buffer (50 mM phosphate buffer pH 7, 500 mM NaCl, 50 mM imidazole, 100 µM ADP, 1 mM MgCl_2_) supplemented with EDTA-free protease inhibitor tablet (Roche) and benzonase (Novagen). *hs*RuvBL2 was obtained through several purification steps: (i) a Nickel affinity column (HisTrap HP, GE Healthcare), by elution with a gradient of buffer containing 1 M imidazole; (ii) an anion exchange column (Resource Q, GE Healthcare), by elution with a gradient of buffer with 1 M NaCl; (iii) a size exclusion column (HiLoad 16/600 Superdex 200 pg, GE Healthcare), previously equilibrated with 20 mM Na/K phosphate pH 8, 200 mM NaCl, 3 mM MgCl_2_ and 500 µM ADP. *hs*RuvBL2 eluted as a hexamer at a concentration of 10 mg/mL.

### Differential scanning fluorimetry (DSF)

The protein melting temperature (T_m_) determination was performed by monitoring protein unfolding with the fluoroprobe SYPRO Orange dye, in an iCycle iQ5 Real Time PCR Detection System (Bio-Rad) equipped with a charge-coupled device (CCD) camera and a Cy3 filter with excitation and emission wavelengths of 490 and 575 nm, respectively. The plates were heated from 20 to 90 °C with stepwise increments of 0.5 °C with a 60-second equilibration time, followed by the fluorescence read out. To test the effect of nucleotides, the protein was incubated with nucleotides with a molar excess higher than 10-fold (4 mM of nucleotide to *ca*. 300 µM *hs*RuvBL2). All assays were performed with pure protein (N = 3), from the peak corresponding to a hexameric oligomer, collected from the last size exclusion purification step.

### Analytical ultracentrifugation (AUC)

AUC analysis of sedimentation coefficients of *hs*RuvBL2 was performed to determine differences in the oligomerization state of the different *hs*RuvBL2 constructs in ADP-containing buffer. Sedimentation velocity (SV) was used to determine the proportion of different oligomeric forms for each sample, as well as their approximate molecular weight, and to assess whether the *hs*RuvBL2 complex undergoes concentration-dependent dissociation. SV experiments were performed at 35000 revs per min and 20 °C, in a XL-I analytical ultracentrifuge using an Anti-50 rotor (Beckman Coulter, USA), with 3 and 12 mm path length Ti double-sector centrepieces equipped with sapphire windows (Nanolytics GmbH, Germany), loaded with 110 and 420 µL, respectively, depending on protein concentration, sample and reference solvent. Acquisitions were made using interference optics. The reference channels were filled with the buffer without ADP. Data analyses were done with the Sedfit software^40^, v14.1, using the program SEDNTERP (http://sednterp.unh.edu/), considering buffer density, *ρ* = 1.025 g mL^−1^ and viscosity, *η* = 1.06 cP. For homogeneous samples, the non-interacting species analysis provides independent estimates of sedimentation coefficient s and of the diffusion coefficient D, which was used in the Svedberg equation s_0_/*D* = M (1 − *ρ*$\bar{v}$)/*RT*, to provide an experimental value for the molar mass, M.

### Small angle X-ray scattering (SAXS)

*hs*RuvBL2 was measured at beamline B21, Diamond Light Source (Didcot, UK; Experiment number SM13193-1), using both static measurement through the sample changer (Maatel, Grenoble) and through in-line size-exclusion chromatography column (SEC-SAXS). Analysis in static mode was performed using Scatter (Bioisis.net) by merging datasets from serial dilutions of a *hs*RuvBL2 fraction from the centre of the S200 peak, from 0.6 to 4.5 mg/mL. SEC-SAXS analysis was performed by injection of a fraction at 5.5 mg/mL from the middle of the S200 peak, onto a Shodex 403 kw column (Supplementary Fig. S6). Measurements were taken each second across the peak elution. Frames that were shown to have the same Rg were selected and scale-merged using Scatter. Further analysis used Scatter and FoXs^41^. All samples were filtered through a 1 MDa filter prior to analysis to eliminate larger aggregates.

### Electrophoretic mobility shift assays (EMSA)

Electrophoretic mobility shift assays were performed to assess *hs*RuvBLs ability to bind ssDNA in native conditions. Prior to the agarose gel separation, the protein was incubated with M13mp18 in a reaction mixture with a total volume of 20 µL, of which 0.5 µL (1.12 nM) DNA, variable amounts of protein (50 or 25 µM) and completed with reaction buffer (25 mM HEPES/KOH pH 8, 2.5 mM Mg(CH_3_COO)_2_, 100 mM KCl, 0.2 mM DTT, 4 mM ATP and 2 mM MgCl_2_). Negative controls were performed by incubating either only DNA or only protein in the reaction buffer. The reaction occurred for 1 h at 23 °C, after which 1 µL of 50% (V/V) glycerol was added and the reaction loaded in a 0.6% (w/V) agarose gel in TBE 1x and run for 2 h30 m at 80 V. The DNA bands were stained with SYBR Gold DNA stain. Fluorescence was detected with a Fuji TLA-5100, using a 473 nm excitation wavelength.

### Analysis of *hs*RuvBL2 binding to DNA by negative staining EM

Immediately prior to EM data collection, monomeric *hs*RuvBL2 was incubated with M13mp18 circular ssDNA. A control incubation was prepared without DNA. After 30 minutes of incubation at room temperature (about 20 °C) in buffer with ATP, both samples (with and without DNA) were adhered to Rhodium/Copper EM grids (pre-treated with a carbon coating and rendered hydrophilic through an electrical glow discharge), washed with Tris-based buffer, and stained with uranyl acetate. Micrographs were obtained using a Tecnai F20 or a JEOL JEM-1010 TEM, with exposure time per picture of 0.2–0.9 s and a dose of 20–25 e^−^/Å^2^ s, and collected using a CCD camera.

### Crystallization and data collection

*hs*RuvBL2 was dialysed to pH 6 immediately prior to crystallization. Crystallization drops were set up at 30 °C using the vapour-diffusion sitting-drop method in 24-well Linbro plates, with a proportion of 0.7 µL of protein to 0.3 µL of a reservoir solution composed of 250 mM MgCl_2_ and 2% PEG 3350. Protein was at 10 mg/mL and the reservoir volume was 500 µL. Crystals appeared within 1 day, grew to a maximum size of about 200 µm × 40 µm (hexagonal needles) and were cryocooled in mother liquor supplemented with 25% glycerol. Diffraction data from a crystal diffracting to 2.89 Å was collected at Proxima-I beamline, at the Soleil synchrotron source (St. Aubin, Paris, France), using a CCD detector (ADSC QUANTUM 315r).

### Structure solution and refinement

Data were indexed and integrated with *XDS*^42^, and the space group was determined with *POINTLESS*^43^ and scaled with *AIMLESS*^44^, all within the *autoPROC* data processing pipeline. Four datasets were collected from the same crystal, in a total of 347 images 1° wide (347° “wedge”). The four datasets were processed individually and scaled together, yielding a data set with high multiplicity in space group *P*6. Data collection and processing statistics are listed in Table 1. Matthews coefficient calculations^45^ indicated the presence of one molecule per asymmetric unit. The 3D structure of *hs*RuvBL2 was solved by molecular replacement using *PHASER*^46^, with *hs*RuvBL1 domains I and III (cropped from the PDB ID 2C9O) as the search model. The total amount of collected images was used to obtain an initial electron density map, making use of the high multiplicity of data, which helped to highlight electron density fragments not clearly visible upon phasing. Electron density was further improved with with *BUSTER-TNT*^47^ making use of the “-L” flag within the program. Iterative cycles of automated model building with *BUCCANEER*^48,49^, within the *CCP4* suite of programs^50^, and refinement with *BUSTER-TNT*, were performed until electron density was clearly visible for domain II. Iterative cycles of manual model building and refinement were subsequently performed with *COOT*^51^ and *BUSTER-TNT*. The program *HYDROGENATE* (distributed with *BUSTER-TNT* which uses the program *REDUCE*^52^ from the *MolProbity* suite), was used to add hydrogen atoms (with zero occupancy), to the crystallographic model during refinement. The final refinement round was performed with *phenix*.*refine* within the *PHENIX*^53^ suite of programs using the data obtained from a 61°-wedge of diffraction images with better integration statistics. Model validation was performed simultaneously with model building and refinement using *MolProbity*^54^ as implemented in *BUSTER-TNT* and *PHENIX*.

### Electrostatic surface calculations

The software *CHARMM*^55^ was used to calculate the surface charges distribution in *hs*RuvBL1 and *hs*RuvBL2. Topological visualization of the electrostatic potential was produced in PyMol^56^ with *APBS*^57^.

## Electronic supplementary material

Supplementary video 1

Supplementary Information

## Data Availability

Coordinates of the refined PDB and structure factors were deposited in the RCSB Protein Data Bank (www.rcsb.org)^58^ with the PDB ID entry 6H7X. All other data are available from the corresponding author on reasonable request.
