# Supplementary material for: X-ray structure of full-length human RuvB-Like 2 – mechanistic insights into coupling between ATP binding and mechanical action
Source: Sci Rep. 2018 Sep 13;8:13726. doi: 10.1038/s41598-018-31997-z (PMC6137109; doi:10.1038/s41598-018-31997-z)
Supplement: Supplementary file 2 — Supplementary Information [file 41598_2018_31997_MOESM2_ESM.docx]

Supplementary Information

X-ray structure of full-length human RuvB-Like 2 – mechanistic insights into coupling between ATP binding and mechanical action.

**Authors:**

Sara T.N. Silva^1,2^, José A. Brito^1^, Rocío Arranz^3^, Carlos Óscar S. Sorzano^3^, Christine Ebel^4^, James Doutch^5^, Mark D. Tully^6^, José-María Carazo^3^, José L. Carrascosa^3^, Pedro M. Matias^1,2^, Tiago M. Bandeiras^2^

^1^ Instituto de Tecnologia Química e Biológica António Xavier, Universidade Nova de Lisboa, Av. da República, 2780-157 Oeiras, Portugal

^2^ iBET, Instituto de Biologia Experimental e Tecnológica, Apartado 12, 2780-901 Oeiras, Portugal

^3^ Department of Structure of Macromolecules, Centro Nacional de Biotecnología (CNB-CSIC), Campus Cantoblanco, 28049 Madrid, Spain

^4^ Institut de Biologie Structurale (IBS), Univ. Grenoble Alpes, CNRS, CEA, 71 avenue des Martyrs CS 10090, 38044 Grenoble, France

^5^ ISIS Pulsed Neutron and Muon Source, STFC, Harwell Science and Innovation Campus, Didcot, OX11 0QX, UK

^6^ European Synchrotron Radiation Facility (ESRF), Grenoble, France


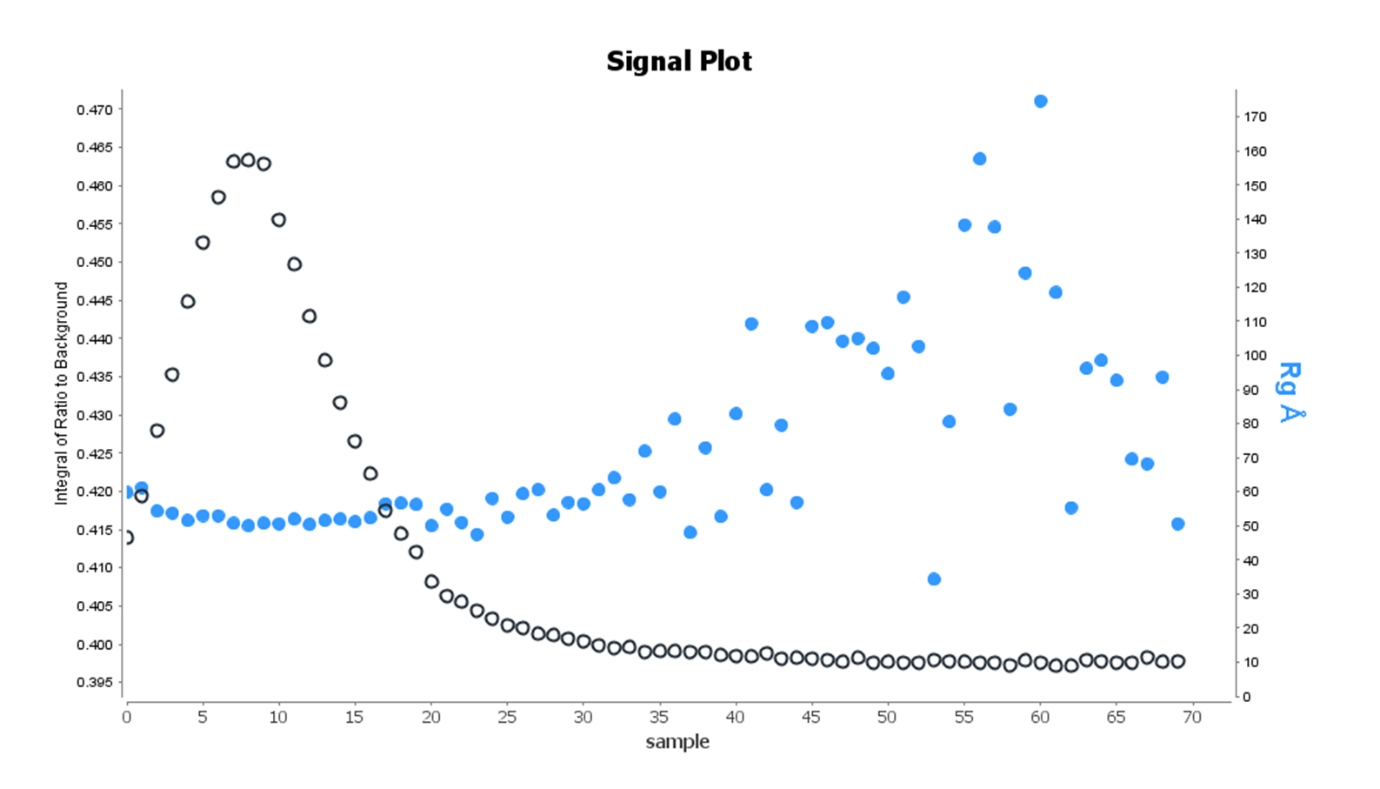


**SM Fig1 Small angle X-ray scattering of *hs*RuvBL2.** In-line analysis across the size-exclusion chromatography elution peak shows that *hs*RuvBL2 has a constant radius of gyration, about 52 Å.


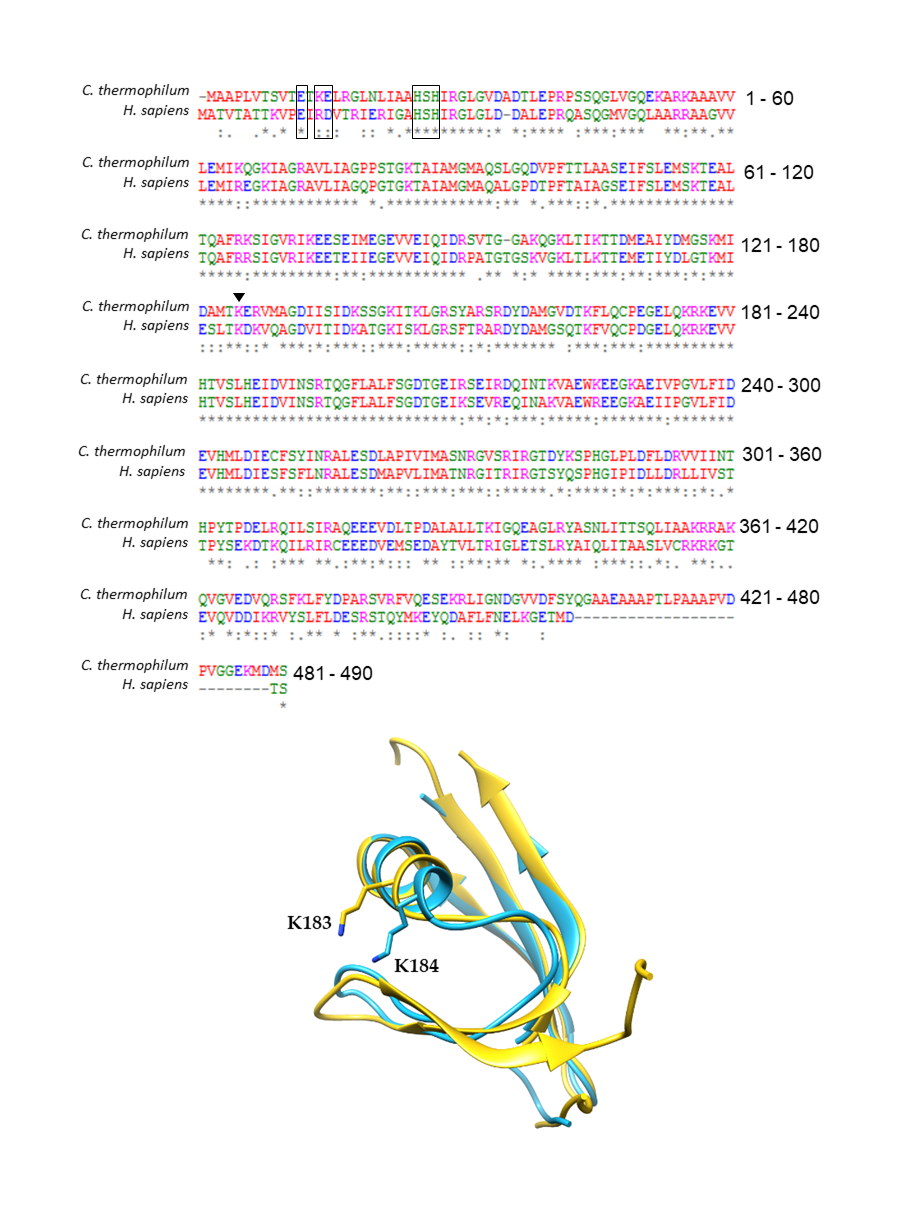


**SM Fig2 Alignments of RuvBL2 from *Homo sapiens* and *Chaetomium thermophilum*.** **Top:** alignment of protein sequences. The highlighted residues are putatively involved in the reorganisation of the N-terminal loop, and the Lysine marked with a triangle is putatively involved in the stabilisation of domain II. **Bottom:** Structure alignment of the OB-folds from the two structures (human 6H7X, cyan and *C. thermophilum* 4WW4, yellow) shows that the residues putatively involved in the stabilisation of domain II have the same spatial distribution. The Lysine residues depicted as sticks have a role in the proposed mechanism, and are conserved between the two organisms (as indicated by a triangle in the top panel). Structure alignment obtained with Chimera.


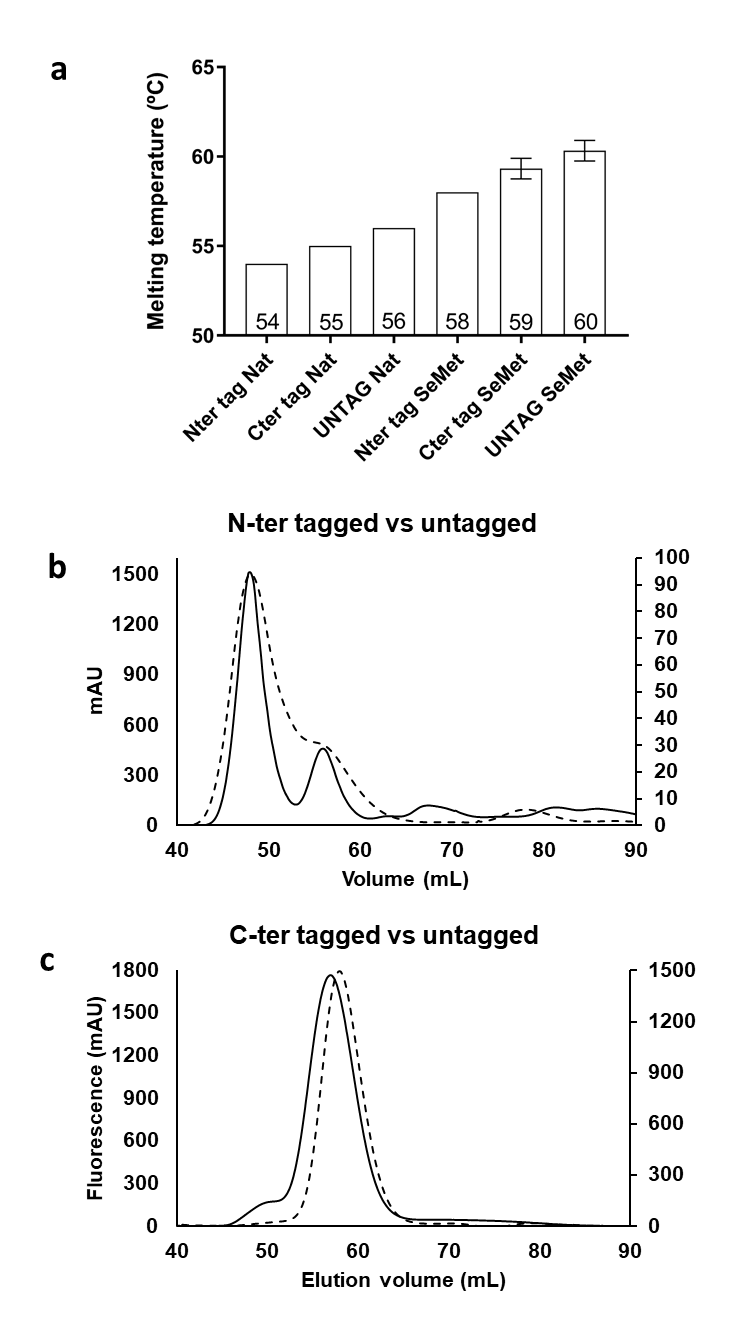


**SM Fig3 Tags influence *hs*RuvBL2 stability and oligomerization plasticity. (a)** Thermal shift assays show that placing a tag on the N-terminus is detrimental to *hs*RuvBL2 stability in solution, both in the native state, and in the selenomethionine-substituted form (tested for crystallization purposes). The most stable construct is obtained after tag cleavage from the C-terminus. **Nter tag Nat** – native form with tag on N-terminus**; Cter tag Nat** - native form with tag on C-terminus; **UNTAG Nat** - native form after tag removal from C-terminus; **Nter tag SeMet** – selenomethionine form with tag on N-terminus**; Cter tag SeMet** - selenomethionine form with tag on C-terminus; **UNTAG SeMet** - selenomethionine form after tag removal from C-terminus. **(b)** When *hs*RuvBL2 is expressed with a tag on the N-terminus, it forms both hexamers and dodecamers, both before (solid line) and after (dashed line) tag removal. However, when the tag is placed on the C-terminus **(c)**, *hs*RuvBL2 forms mostly hexamers (tagged *hs*RuvBL2 - solid line), an oligomeric state also maintained after tag removal (dashed line).


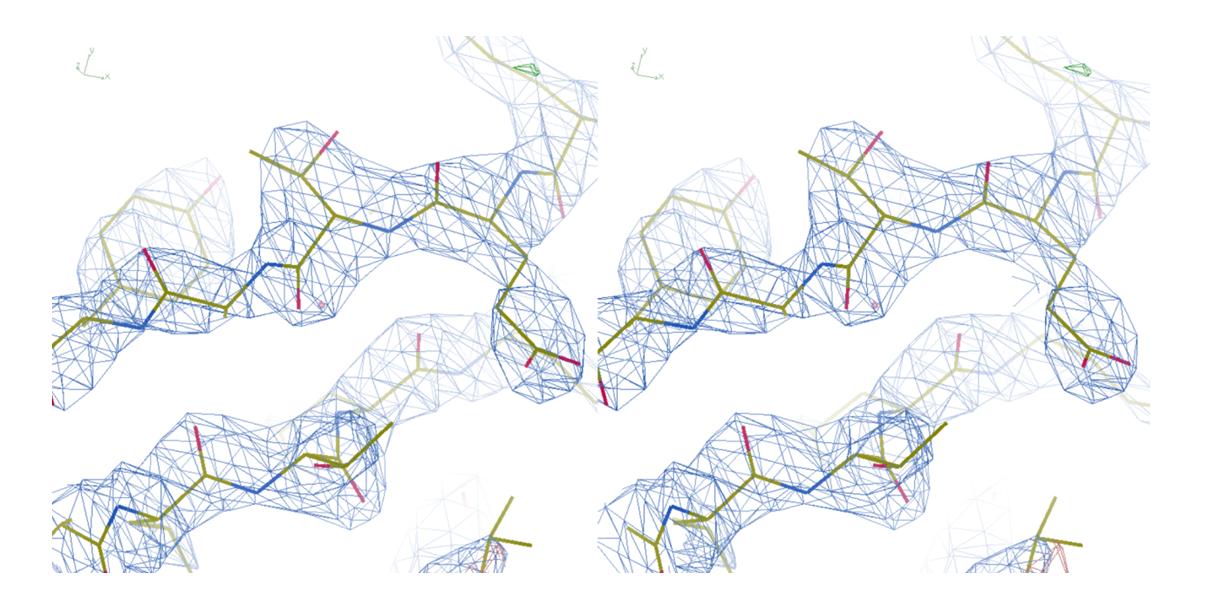


**SM Fig4 Stereo image of the electron density of *hs*RuvBL2.**


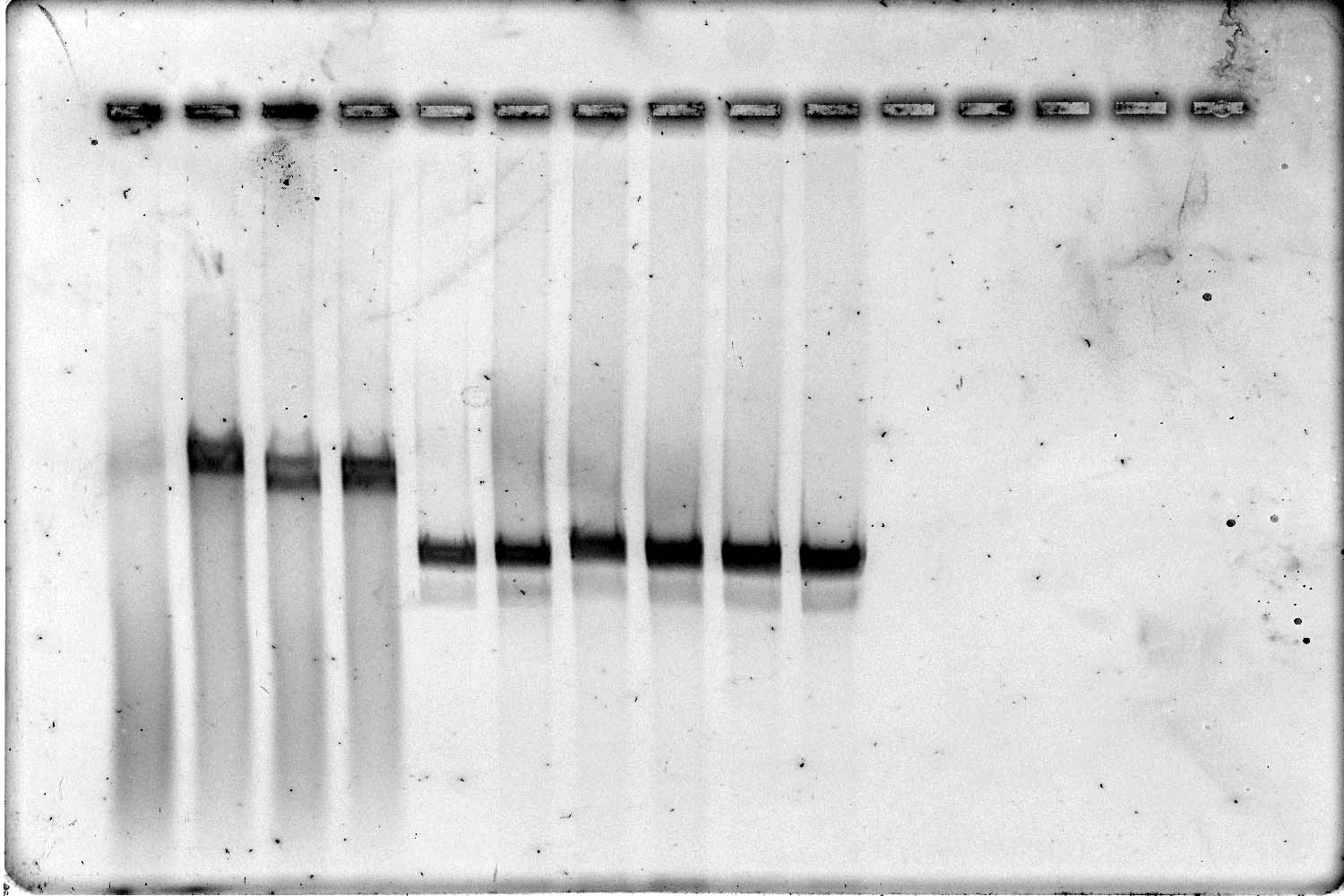


R1 R1 R1 --- R2 R1A R1A R1A R1A

R2 R2 R2 R2

µM 50 25 25 50 50 50 25 25

M13mp18

+

**SM Fig5 Complete agarose gel from fig. 6.**

**SM Fig4 RuvBL2 is able to form heptamers.** 10% of the total of picked particles corresponded to this oligomeric form, highlighted in red.

**SM Fig4 RuvBL2 is able to form heptamers.** 10% of the total of picked particles corresponded to this oligomeric form, highlighted in red.
